# Fungal diversity differences in the indoor dust microbiome from built environments on earth and in space

**DOI:** 10.1038/s41598-024-62191-z

**Published:** 2024-05-24

**Authors:** Nicholas Nastasi, Sarah R. Haines, Ashleigh Bope, Marit E. Meyer, John M. Horack, Karen C. Dannemiller

**Affiliations:** 1 Environmental Science Graduate Program, Ohio State University, Columbus, OH 43210 USA; 2 Department of Civil, Environmental and Geodetic Engineering, College of Engineering, Environmental Health Sciences, The Ohio State University, 470 Hitchcock Hall, 2050 Neil Ave, Columbus, OH 43210 USA; 3 Division of Environmental Health Sciences, College of Public Health, Ohio State University, Columbus, OH 43210 USA; 4 Department of Civil and Mineral Engineering, University of Toronto, Toronto, ON Canada; 5 NASA Glenn Research Center, Cleveland, OH 44135 USA; 6 Department of Mechanical and Aerospace Engineering, College of Engineering and John Glenn College of Public Affairs, Ohio State University, Columbus, OH 43210 USA; 7 Sustainability Institute, The Ohio State University, Columbus, OH 43210 USA

**Keywords:** Environmental microbiology, Fungi

## Abstract

Human occupied built environments are no longer confined to Earth. In fact, there have been humans living and working in low-Earth orbit on the International Space Station (ISS) since November 2000. With NASA’s *Artemis* missions and the age of commercial space stations set to begin, more human-occupied spacecraft than ever will be in Earth’s orbit and beyond. On Earth and in the ISS, microbes, especially fungi, can be found in dust and grow when unexpected, elevated moisture conditions occur. However, we do not yet know how indoor microbiomes in Earth-based homes and in the ISS differ due to their unique set of environmental conditions. Here we show that bacterial and fungal communities are different in dust collected from vacuum bags on Earth and the ISS, with Earth-based homes being more diverse (465 fungal OTUs and 237 bacterial ASVs) compared to the ISS (102 fungal OTUs and 102 bacterial ASVs). When dust from these locations were exposed to varying equilibrium relative humidity conditions (ERH), there were also significant fungal community composition changes as ERH and time elevated increased (Bray Curtis: R^2^ = 0.35, P = 0.001). These findings can inform future spacecraft design to promote healthy indoor microbiomes that support crew health, spacecraft integrity, and planetary protection.

## Introduction

In the upcoming decades, human activity in space will rapidly expand both in low-Earth orbit (LEO) and beyond to an estimated $1.4 trillion dollar industry^1^. NASA’s *Artemis* missions, which will send humans back to the moon, includes a lunar orbiting space station (The Lunar Gateway) as well as a lunar surface habitat, and will all be a stepping stone to sending humans to Mars^2^. In addition, private companies, such as Starlab Space, LLC and Blue Origin, are building commercially owned and operated space stations with support from NASA’s commercial LEO destination initiative^3^. The rapid increase in the number of people living and working in space will require careful spacecraft design to support human health and wellbeing. One critical aspect of this design is to understand the microorganisms within a spacecraft.

The International Space Station (ISS) has been a continuously inhabited confined environment in LEO since November 2000, and over 12,000 microbial species have been identified onboard^4^. The ISS is a closed system and the microbes primarily originate from the astronauts onboard^5^, as all humans have a distinct associated microbial ecosystem^6^. Other potential microbial sources on the ISS include plant experiments being grown onboard^7^, as well as those from the original construction materials. The ISS has its own unique microbiome, as does every human-occupied indoor environment on Earth.

Similar to Earth-based indoor environments^8^, many of these microbes can reside in the dust generated onboard the ISS^9^. This is due to the high nutrient content in dust that microbes can utilize, with moisture being the limiting factor for growth^10,11^. Bacterial communities from ISS dust and other Earth-based confined environments such as clean rooms, operating rooms, and intensive care units each contain a similar core microbial community^12^, although there are some significant differences between environment types^13^. Whether in space or on Earth, it is critical to maintain microbial communities indoors that support human health^14^. For instance, health effects on Earth are associated with microbial growth^15^, and thus it is critical to prevent this growth by controlling indoor moisture. Although, NASA standards for nominal relative humidity conditions on the ISS is between 25 and 75%^16^, there are pockets of elevated moisture present due to astronaut hygiene, exercising, in food areas, and in plant habitats. This study also provides insight into how microbial communities will react to unintended conditions such as a ventilation system failure or fire suppression event. There have also been reported issues reported on the ISS due to unintended microbial growth such as crewmember infections^17^ and destruction of plants onboard^18^. However, we need an improved understanding of how the microbial communities on the ISS compare to typical occupied spaces on Earth, and how these communities change under unexpected adverse conditions such as excess moisture.

The goal of this study is to determine similarities and differences between microbial communities and growth under elevated moisture conditions in occupied spaces on Earth and the ISS. Here, we leverage raw sequencing data from two previous studies with nearly identical methods that have performed microbial analyses on dust collected from the ISS^19^ and from Earth-based residential homes^20^. These studies have a special focus on indoor fungi and characterize what happens when ideal growth conditions occur such as the introduction of elevated moisture conditions. This is a unique one-to-one comparison as methods were performed in the same laboratory, therefore all incubation, DNA extraction, DNA sequencing, and bioinformatics protocols are almost identical. Key differences between the studies were the presence of dust in carpet for Earth-based samples and different starting dust mass concentrations (50 mg for Earth-dust and 25 mg for ISS dust).

The ISS is a complex environment that serves as an astronaut home, workplace, and transportation environment. While it can’t be distinctly classified as only one of these, we chose the residential home comparison because people on Earth spend most of their time in their home and to emphasize that astronauts are spending all their time in the spacecraft, including during sleep. This comparison of Earth- and space-based microbiomes in continuously occupied indoor environments will help us gain a better understanding of how the unique conditions experienced during spaceflight may alter microbial communities. As humanity becomes more active in space, the need to define what a healthy microbiome is in human occupied spacecraft will grow ever more important. These results can inform both Earth-based and future space-based indoor spaces on how microbial communities may change to identify any potential problems that may arise when unintended microbial growth occurs in these environments.

## Results

### Fungal communities in original dust samples

**Figure 1:**
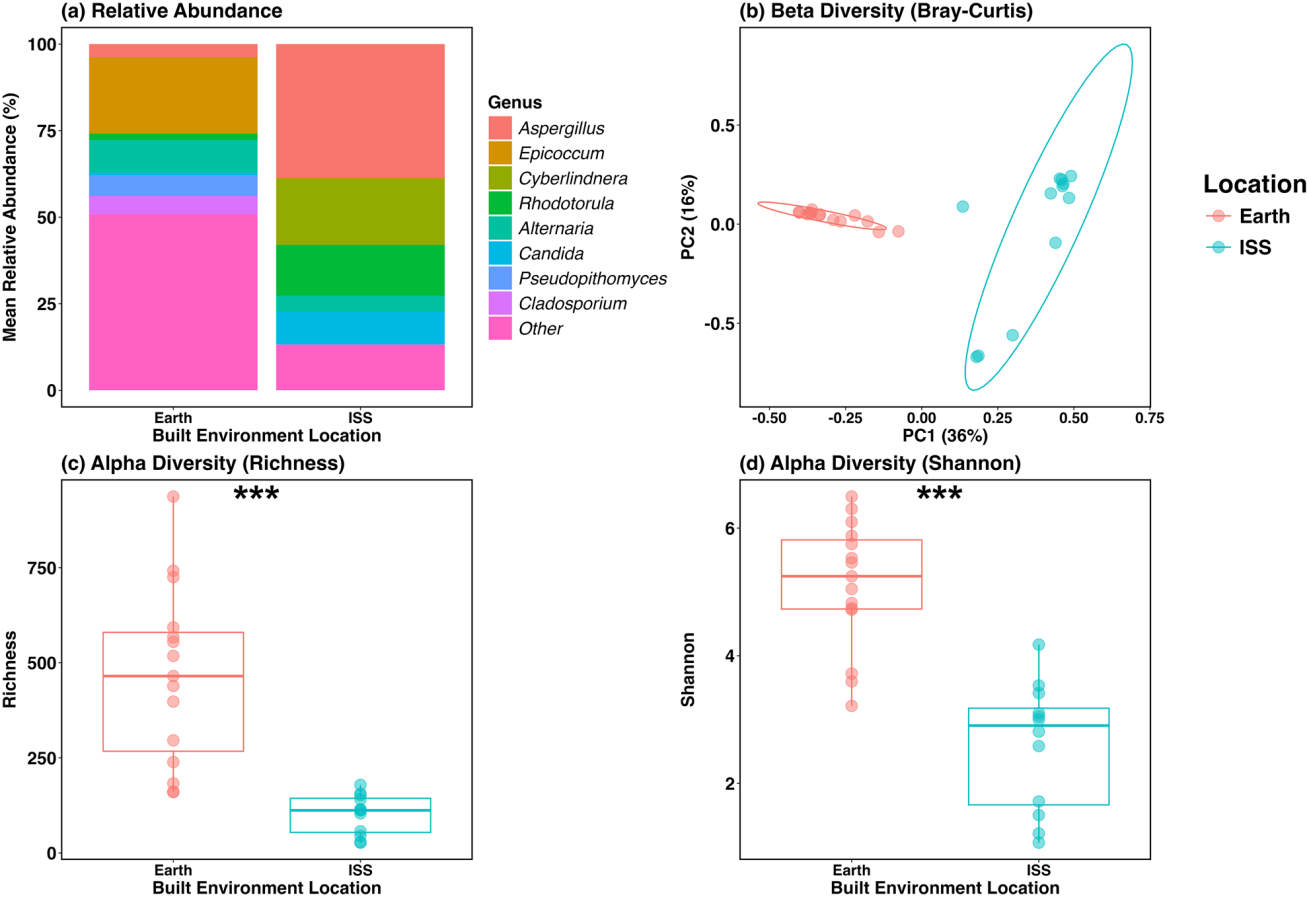
Fungal comparisons of original dust (no incubations or ERH exposure) from Earth-based residential homes and the International Space Station. (**a**) Relative abundance of fungal genera comparison of these locations with “Other” being any fungal genera that was below 5% relative abundance cumulatively. (**b**) Fungal beta diversity using the Bray–Curtis dissimilar matrix show a compelling difference between Earth-based and ISS samples. Ellipses represent the 95% confidence interval. Fungal (**c**) Richness and (**d**) Shannon diversity plots comparing original dust from Earth-based residential homes to dust from the International Space Station. For all plots there was a total of 15 Earth-based original dust samples (1 for 15 residential homes) and 4 ISS dust bag samples (that included triplicates for each bag). ***Indicates Kruskal–Wallis t-test significance of P < 0.0001.

We compared fungal communities in dust from the ISS and Earth-based residential homes. These samples are referred to as “original dust” and are the dust collected from vacuum bags from both locations that were not exposed to any elevated equilibrium relative humidity (ERH) conditions in the laboratory prior to analysis. In total we had 15 original dust samples from Earth (1 sample each from 15 separate homes) and 4 original dust samples from the ISS (which included 3 triplicates from a total of four separate ISS vacuum bags). The most abundant fungal genera from these locations were different. *Epicoccum, Alternaria, Pseudopithomyces,* and *Cladosporium* were the dominant fungal genera on Earth while *Aspergillus, Cyberlindnera, Rhodotorula,* and *Candida* were more abundant on the ISS (Fig. 1a).

Diversity metrics were analyzed using operational taxonomic units (OTUs) for fungi and amplicon sequence variants (ASVs) for bacteria due to the differences in bioinformatic pipelines used in this study. For original dust, fungal beta diversity significantly separated out by Earth and ISS samples (R^2^ = 0.23, P = 0.001, Table S1) (Fig. 1b). In general, the Earth samples were much more diverse compared to ISS samples. Earth original dust samples had a mean fungal OTU count of 465, while the ISS dust had a mean of only 102 OTUs (Table S2). Alpha diversity also showed significant differences in Richness and Shannon diversity metrics when comparing original dust samples between these locations (Richness H = 18.44, P < 0.0001; Shannon H = 17.20, P < 0.0001) (Fig. 1c,d). We also performed a differential absolute abundance analysis on original dust samples showing there were a total of 791 fungal species identified with 119 more abundant in Earth-based dust from a wide variety of genera and 24 species more abundant in ISS samples (primarily from the *Aspergillus*, *Malassezia*, and *Penicillium* genera) (Table S3).

### Fungal growth in dust is also different under elevated moisture conditions

We compared growth in fungal communities under varying elevated moisture conditions between Earth and the ISS. For each study, the same incubation procedure was followed in which dust (for ISS), or dust/carpet (for Earth) were incubated at 50% ERH and then exposed to 85% or 100% ERH for 0-, 6-, 12-, 18-, and 24-h per day. In this study, we compared incubated samples for each varying ERH condition (85% and 100% ERH) by combining all time points (Days 5, 10, and 14) and averaging by location (4 Earth-based homes and 4 ISS vacuum bags) as well as original dust samples.

**Figure 2:**
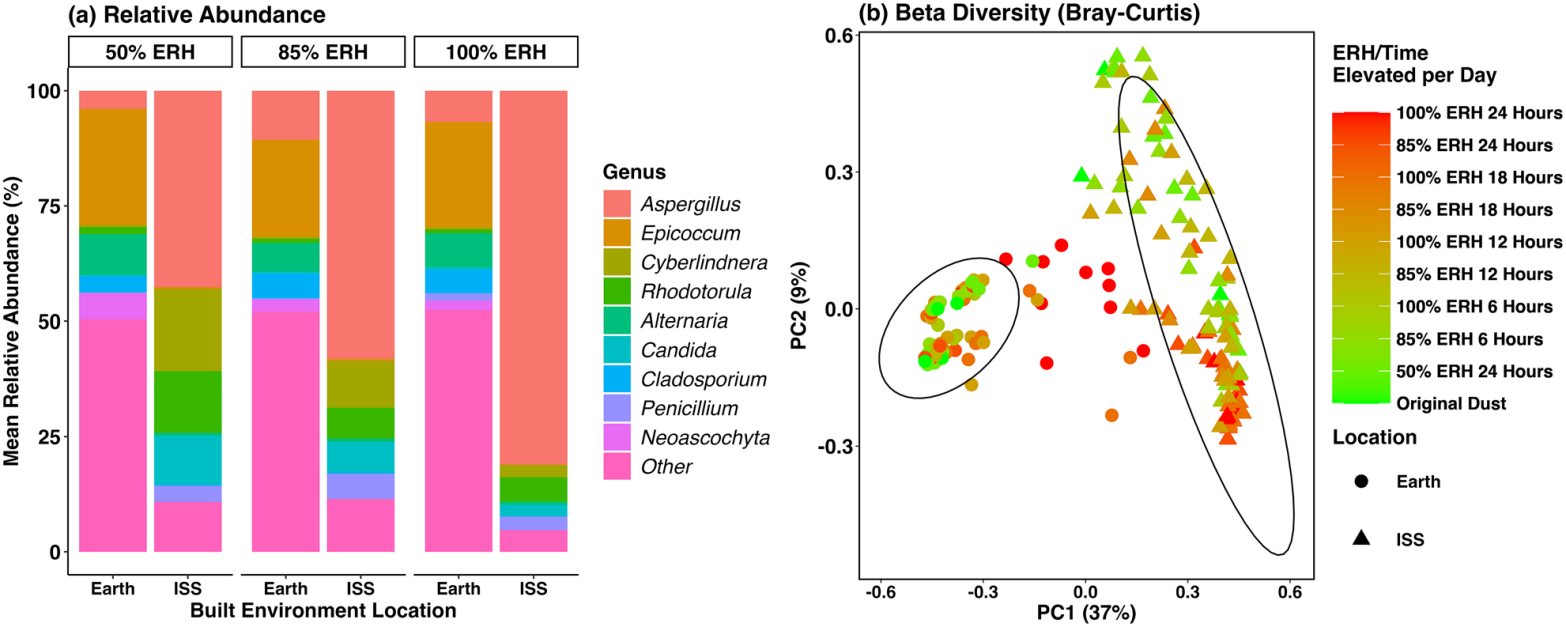
Fungal comparisons of varying equilibrium relative humidity (ERH) samples from Earth-based residential homes and the International Space Station. For both plots Earth and ISS samples represent the average of species measured in days 5, 10, and 14 combined for each relative humidity condition. (**a**) Relative abundance of fungal genera comparison of these locations with “Other” being any fungal genera that was below 5% relative abundance cumulatively. (**b**) Fungal beta diversity using the Bray–Curtis dissimilar matrix show a compelling difference between Earth-based and ISS samples. Ellipses represent the 95% confidence interval. For both plots there was a total of 108 Earth-based original dust samples and 107 ISS samples.

Fungal relative abundance for all ERH conditions (50%, 85%, and 100%) were similar to original dust samples with *Epicoccum* dominating for Earth samples and *Aspergillus* in ISS samples. However, when comparing all varying ERH samples, *Aspergillus* outcompetes all other fungal genera in ISS samples, where the communities in Earth samples remain relatively stable (Fig. 2a). Fungal communities of all combined varying ERH samples also varied based on location (R^2^ = 0.35, P = 0.001) especially at lower ERH conditions and shorter times elevated, with communities from different locations becoming more similar as ERH/time elevated increased (Fig. 2b). Additional breakdowns for fungal beta diversity at specific ERH conditions (50%, 85%, and 100%) and times elevated (6-, 12-, 18-, and 24-h) all showed fungal community composition differences between Earth and ISS samples (Fig. S1, Table S1).

**Figure 3:**
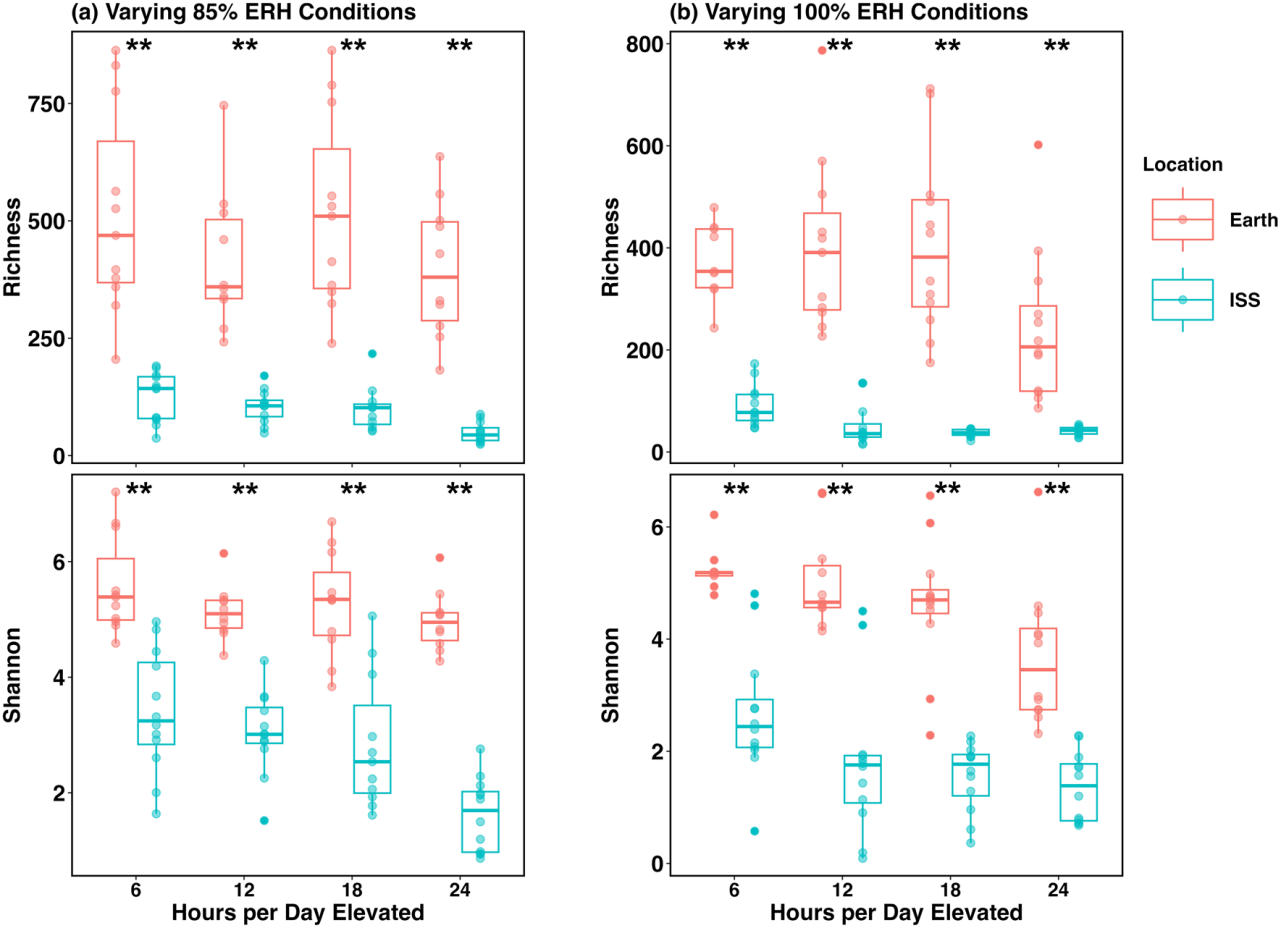
Fungal alpha diversity plots of time-of-wetness samples for Earth-based residential homes (red) and from the International Space Station (blue). For both plots Earth and ISS samples represent the average of species measured in days 5, 10, and 14 for each relative humidity condition. Comparisons show Shannon diversity and Richness metrics for both (a) 85% and (b) 100% ERH conditions and the time fraction per day elevated on x-axis. ** Indicates Kruskal–Wallis t-test between Earth and ISS samples for all times.

Fungal alpha diversity was also significantly different between Earth and ISS varying ERH samples at 85% and 100% ERH for all times elevated (Kruskal–Wallis P < 0.001 for all conditions) (Fig. 3, Table S4). Earth-based samples were more diverse than ISS dust for all conditions and both locations generally became less diverse as ERH and time elevated increased. For all time points at varying elevated conditions (85% ERH), on Earth there was an average of 462 OTUs while on the ISS there were an average of 95 OTUs (Table S2). This was also true for all times elevated above saturated conditions (100% ERH), as on Earth there was an average of 356 fungal OTUs while on the ISS there were 56 (Table S2). A more detailed breakdown of fungal alpha diversity at each time elevated can be found in Figures S2-S5 and Table S2,S4. We also compared differential absolute abundance for varying ERH samples at 100% ERH and all times elevated (6-, 12-, 18-, and 24-h). For these conditions, a total of 521 fungal species were identified with 456 more abundant in Earth-based house dust and 26 of them being more abundant in ISS dust (Table S5).

### Bacterial community composition also different between earth and ISS samples

**Figure 4:**
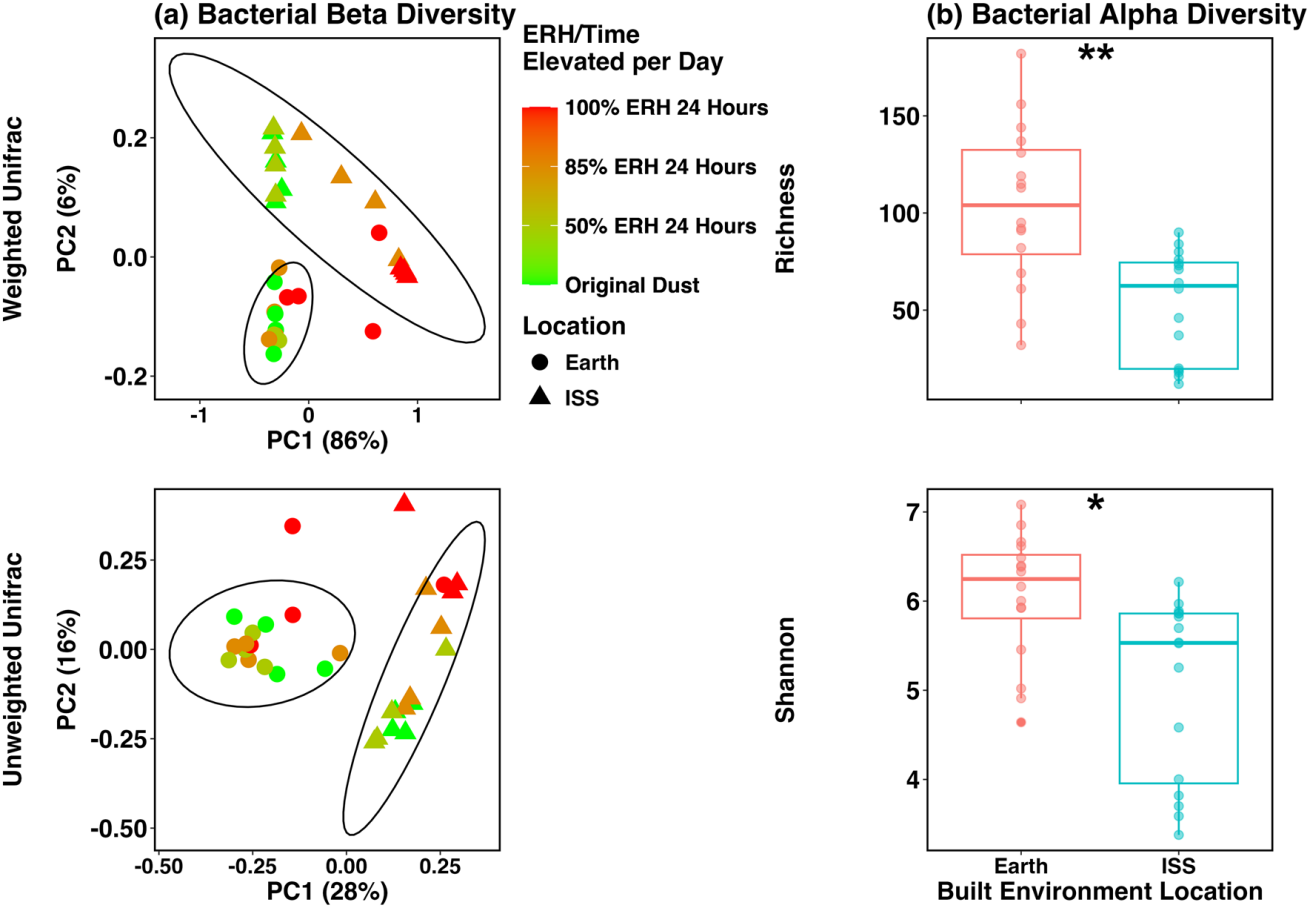
(**a**) Bacterial beta diversity with Bray Curtis dissimilarity principal coordinate analyses plots for original time of wetness samples (TOW). Location of dust samples are indicated by shape (Earth – circle, ISS – triangle) and TOW is indicated by color gradient (Green – Original Dust to Red – 100% relative humidity). Ellipses represent 95% confidence interval for each set of data. (**b**) Bacterial Richness and Shannon diversity plots for time of wetness samples comparing Earth-based house dust to dust collected from the ISS. A total of 4 Earth-based house sites were compared to 4 separate ISS vacuum bags. Only original dust samples and day 14, 24-h time-of-wetness conditions were sequenced for bacteria at 50%, 85%, and 100% RH conditions for both plots. * Indicates Kruskal–Wallis t-test significance of P < 0.05 while ** is P < 0.001.

We compared bacterial diversity in a smaller subset of samples that included original dust from 4 Earth-based residential homes and 4 separate ISS vacuum bags. Bacterial beta diversity for original dust samples visually separated by location, but due to low sample size statistical significance was not able to be determined (Fig. S6). Bacterial diversity in original dust samples was significantly different between Earth and ISS for Richness metrics (H = 5.33, P = 0.021), but not for Shannon diversity metrics (H = 3.00, P = 0.083) (Fig. S7, Table S6). Bacterial diversity was much higher on Earth with 237 bacterial ASVs compared to 102 on the ISS (Table S2). In varying ERH samples, bacterial comparisons we were made using original dust as well as day 14 samples at 50%, 85%, and 100% for 24-h time elevated which included 20 samples from both locations. Bacterial beta diversity for these samples was significantly different between Earth and the ISS for both weighted (R^2^ = 0.16, P = 0.004) and unweighted (R^2^ = 0.22, P = 0.001) unifrac distance matrices (Fig. 4a, Table S7).

Bacterial alpha diversity was also significantly different for time-of-wetness samples between these locations for both Richness (Richness H = 11.38, P = 0.0001) and Shannon diversity (H = 9.79, P = 0.0018) (Fig. 4b, Table S6). Again, for these samples Earth samples were more diverse with 104 bacterial ASVs while only 53 in ISS dust (Table S2). A limited bacterial differential abundance analysis was performed on original dust samples only. Of these samples a total of 81 bacterial species were identified of which 3 species were more abundant in ISS dust (*Acinetobacter rhizosphaerae, Prevotella melaninogenica,* and *Rothia dentocariosa*) and 11 species more abundant in Earth-based dust (Table S8).

**Figure 5:**
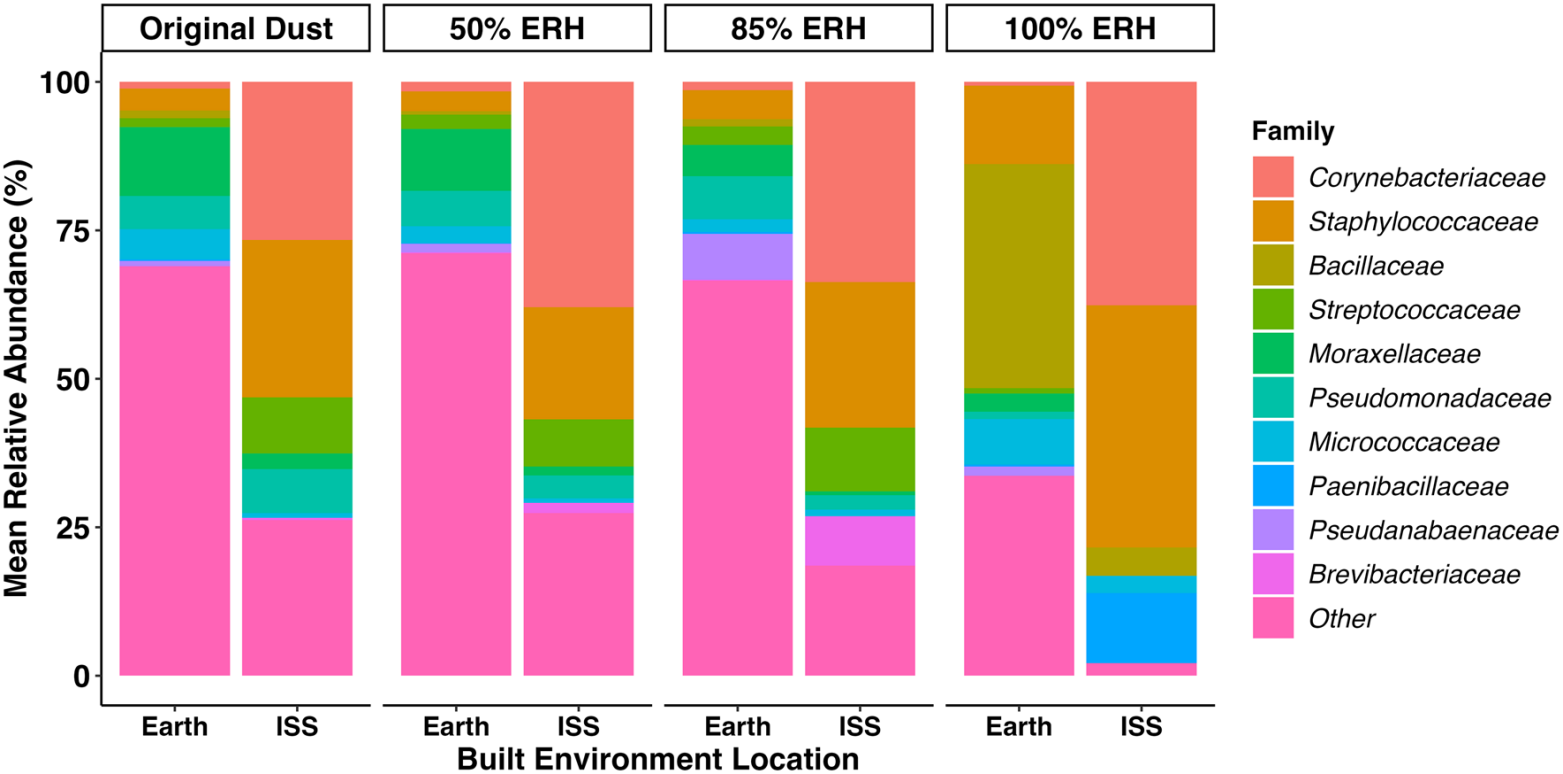
Bacterial family level relative abundance plots comparing Earth- and space-based communities. Original dust samples were the average of 4 Earth-based samples from 4 separate homes and dust from 4 separate ISS vacuum bags. 50%, 85%, and 100% ERH samples were from day 14 only and were also the average of 4 Earth-based homes and 4 ISS vacuum bags.

We also performed a bacterial family level analysis for original dust samples and day 14 time-of-wetness samples. Relative abundance analyses show that for all conditions Earth-based dust and ISS dust contain different bacterial communities (Fig. 5). It also shows that while the communities are different between the two locations, the communities in both remain stable up until the 100% ERH condition is reached, at which point, clear differences can be seen. For Earth-based communities *Moraxellaceae*, *Pseudomonadaceae*, and *Micrococcaceae* were the most abundant families in original dust, at 50% ERH, and at 85% ERH, with *Staphylococcaceae* and *Bacillaceae* becoming most abundant at 100% ERH. For ISS samples *Corynebacteriaceae, Staphylococcaceae*, *Streptoccoceae*, and *Pseudomonadaceae* are most abundant bacterial families up until 100% ERH, when *Corynebacteriaceae* and *Staphylococcaceae* significantly increasing as well as *Paenibacillaceae* becoming a major portion of the community.

## Discussion

There are clear differences in bacterial and fungal communities in dust collected from Earth-based residential homes and from the ISS. This includes original dust immediately removed from a vacuum bag as well as after this dust is exposed to varying elevated moisture conditions to encourage rapid microbial growth. In general, these difference were more pronounced in fungi, especially under elevated moisture conditions, presumably because fungi are more sensitive to changing moisture conditions when compared to bacteria^10,11,20^. Interestingly, fungal communities in ISS dust changed much more rapidly compared to Earth house dust with varying moisture conditions mainly due to *Aspergillus* dominating in the ISS dust. These differences may be attributed to the unique stressors faced by microbes during spaceflight conditions such as microgravity^21^, low-dose radiation^22^, and elevated CO_2_ concentrations^23^ which allows them to rapidly grow when ideal conditions are introduced. In Earth-based house dust the presence of *Epicocum sp.* was the major difference in fungal genera when comparing to ISS dust. This is most likely due to *Epicocum sp.* mostly being associated with soils, fruits, and grains^24^, which are not found in the ISS environment.

The microbes in these locations likely differ due to different environmental contributions. For example, on Earth the number of human occupants^25^, presence of pets^26^, types of building materials, types of ventilation system^27^, and contribution of the outdoor environment^28^ can all have a profound impact on the microbes present. This study shows that Earth-based indoor environments are much more diverse compared to the ISS, which has fewer occupants, no pets (although mouse experiments are often onboard), no outdoor contributions, and different types of materials and ventilation. In addition, behaviors such as cleaning habits also contribute to microbial presence and, on the ISS, cleaning is done weekly with different types of cleaners, none of which are aerosol disinfectants. Earth-based homes can vacuum, use aerosols, carpet powders, and many other products, though we do not know the cleaning habits used in these specific samples and if it could contribute to the differences in the microbial communities.

This study is subject to several limitations, which mainly relate to the location of incubations, as ISS dust was incubated on Earth and not in space. Future research might evaluate how these changes may differ when incubations occur under microgravity, higher radiation levels, and other conditions unique to space travel. In addition, Earth dust samples were also incubated embedded in carpet and vacuumed again before molecular analysis, while ISS samples were not, which could alter the overall results. The different studies had a difference in starting dust mass of 25 mg for ISS and 50 mg for Earth samples and the ISS dust was not sieved, although we do not anticipate that this had a measurable impact on the findings. In addition, not sieving the ISS dust is potentially more representative of dust exposure in the ISS built environment due to lack of gravitational forces settling larger particles. Furthermore, we had limited information of ISS dust sampling, including locations onboard, how long between cleaning events, or if the vacuum was used to clean other areas of the spacecraft. This study utilized a DNA-based analysis that could not determine viability of organisms and is also subject to standard limitations of qPCR and DNA sequencing analysis of microbes^29,30^.

Overall, the microbiome of dust from Earth is more diverse than that of the ISS. These community differences result in changes in microbial growth upon exposure to moisture that may be associated with health impacts. With the ISS being decommissioned in 2030, there will be newly designed commercial space stations taking its place. In addition, there will be more people than ever living and working in space, including the general public with space tourism. This study shows that there are clear differences in a highly regulated government owned and operated spacecraft and these changes can become increasingly important as new station designs, with larger, more varied crews, and commercial objectives launch into low-Earth orbit. This understanding can help us to maintain a healthy indoor microbiome on spacecraft during both increased activity in low-Earth orbit and long duration human-occupied space travel, to promote crew health^17^, spacecraft integrity^31^, and planetary protection^32^.

## Methods

### Overview

This study aims to understand the differences between the indoor microbiomes of Earth-based residential buildings and dust collected from the International Space Station (ISS). Specifically, ISS dust and residential carpet with dust previously collected from homes in Ohio were incubated under varying relative humidity conditions using the time-of-wetness framework^19,20^. Fungal and bacterial DNA was extracted from these incubated samples, sequenced with Illumina MiSeq, and analyzed with a bioinformatics pipeline to determine taxonomy.

### Dust samples

All ISS dust used in this study was obtained from vacuum bags (CELOC hypo-allergenic filter system Oreck# PKBB12DW) from the vacuum onboard which the astronauts use to clean the HEPA filter coverings that are part of the air ventilation system. Briefly, ISS dust samples were triple sealed in plastic bags until they could be returned to Earth, where all experiments were conducted. A detailed breakdown of ISS dust sample collection dates can be found in our previous study^19^. Earth carpet and dust samples were collected in residential homes throughout Ohio, USA in a previous study^20^. Briefly, dust was collected from the homes vacuum bags and carpet was removed directly from the same homes. The dust was embedded into carpet collected from the same home using ASTM method F609-13 after which incubations were then performed. After incubations, the dust was vacuumed from the carpet using Eureka Mighty Mite with adapter for a 19 mm × 90 mm Whatman cellulose thimble. The previous study had sampled 19 residential homes and for comparison to ISS dust, 4 of these homes were randomly selected to correspond to the 4 total ISS vacuum bags collected.

### Incubations

For both studies, incubations occurred in a sterilized 3.8 L glass chamber and placed in a VWR incubator (Model TFFU20F2QWA Radnor, PA USA) set to 25°C covered with parafilm to retain ERH conditions and allow CO_2_ to escape. Salt solutions were made to simulate 50% and 85% ERH conditions, while Deionized (DI) water was used for 100% ERH conditions^19,20^. Onset^®^ HOBO^®^ Data loggers (Onset Computer Corporation, Bourne, MA USA) to monitor ERH and temperature conditions inside the incubation chamber. ISS Dust samples were not sieved and measured out into approximately 25 mg portions that were placed on sterile aluminum foil on a plastic dish^19^. In comparison, Earth dust samples were sieved to 300 μm and embedded in 10 cm × 10 cm carpet squares for incubation. We do not anticipate any significant impact with the carpet and ISS dust as they are very similar in structure due the linty, fibrous nature of the ISS dust. Earth and ISS samples were exposed to varying elevated ERH conditions using the time-of-wetness framework previously outlined^20^, which refers to the time fraction per day that relative humidity conditions are above the 80% threshold^33^. Briefly, samples were incubated between 50% ERH and elevated (85% ERH)/saturated (100% ERH) conditions for 0, 6, 12, 18, and 24 h per day. ISS Samples were extracted and quantified at day 5, 10, 14, and 21 after incubation (one 25 mg dust sample per day per bag)^19^, while Earth samples were analyzed at day 0, 5, 10, and 14 after vacuuming approximately 50 mg of dust from carpet squares^20^.

### DNA extraction and sequencing

DNA was extracted from dust samples for both studies using a DNeasy Powerlyzer Power Soil Kit (Qiagen, Hilden, Germany) with a modified bead mixture^34^ (1 g garnet, 0.3 g 100 µm glass beads, and 0.1 g 500 µm beads). Each DNA extraction run included a blank and was confirmed to contain no microbial DNA. A total of 50 µL of DNA extract was collected for each sample and stored at -20℃ until use. Sequencing for all samples was performed on an Illumina MiSeq™ at RTL Genomics (Lubbock, TX, USA). For fungal sequencing ITS1F (CTTGGTCATTTAGAGGAAGTAA) and ITS2aR (GCTGCGTTCTTCATCGATGC) ribosomal DNA primers^35^, while bacteria used 515F (5’-GTGCCAGCMGCCGCGGTA) and 806R (5’-GGACTACHVHHHTWTCTAAT) primers with 2 × 300 bp sequencing reads.

Differential abundance analyses used total fungal and bacterial quantitative polymerase chain reaction (qPCR) values measure at the kingdom level as previously described^11,20^. To quantify fungal concentration, we targeted the 18S rRNA gene with forward primer (FF2) 5′-GGTTCTATTTTGTTGGT TTCTA-3′, and reverse primer (FR1) 5′-CTCTCAATCTGTCAATCCTTATT-3′^36^. For bacteria we targeted the 16S rRNA gene with forward primer 5’-TCCTACGGGAGGCAGCAGT-3’, reverse prime 5’-GGACTACCAGGGTATCTAATCCTGTT-3’, and PROBE (6-FAM)-5’CGTATTACCGCGGCTGCTGGCAC-3’-(BHQ)^36–38^.

For the purposes of this study, we compared a randomly selected 4 out of 19 sites from the Earth-based study to the 4 ISS bags for varying ERH comparison and included samples from the original dust as well as at day 5, 10, and 14 for all ERH conditions. For original dust samples, all available samples were used which included a total of 15 samples (one per home as available) from the Earth-based study compared to 4 total ISS samples (with three replicates per ISS vacuum bag).

### Bioinformatics

The raw sequencing files were extracted from both previous studies at NASA’s GeneLab database (OSD-694, GLDS-623) (for ISS samples) and the European Nucleotide Archive under accession number PRJEB37053 (for Earth samples). All sequences were then combined and run together using Quantitative Insights Into Microbial Ecology 2 (QIIME2), version 2021.8, bioinformatics pipeline to analyze raw FASTQ sequencing data. A note that Earth samples from the original study were originally analyzed using the QIIME1, version 1.9^20,39^, but re-analyzed using QIIME2 for this comparison. For both fungi and bacteria, primers and spacers were trimmed using the Cutadapt plug-in^40^ and paired-ends joined using the VSEARCH join-pairs method^41^. Sequences were then quality trimmed to a Phred score of 30 with a 3 low-quality base window using the Quality-filter plugin^42^.

Fungal sequences were clustered using the VSEARCH open reference method with Unite database reference sequences (version 9.0)^43,44^, while bacterial sequences were quality checked using Dada2^45^ and clustered by phylogeny. Beta Diversity metrics for fungi were analyzed using Bray–Curtis dissimilarity while bacteria utilized unweighted and weighted unifrac statistics with a principle coordinate analysis (PCoA) and Adonis function in QIIME2. Alpha diversity metrics were calculated for all incubations and significance (P < 0.05) determined using the Kruskal–Wallis test statistic from QIIME2.

The Basic Local Alignment Search Tool (BLAST) version 2.9.0^46^, the User-friendly Nordic Internal Transcribed spacer Ectomycorrhiza (UNITE) 2022 database^44^, and Fungal High-throughput Taxonomic tool for use with Next-Generation Sequencing (FHitINGS) version 1.4^47^ was used to identify fungal taxonomy. Bacteria utilized the Greengenes database version 13_8^48^ in QIIME2 using the feature-classifier plugin^43^.

### Statistical analyses

Principal Coordinate Analysis (PCoA) plots using Bray–Curtis and UniFrac distance matrices were created in QIIME2 version 2021.8.0 then imported into R Studio (version 2021.09.0 Build 351) for visualization. The Adonis function was used to significance of beta diversity between Earth- and ISS-based dust samples and time elevated at each relative humidity condition. Alpha diversity significance was determined using a Kruskal–Wallis test, comparing by relative humidity condition and location. We also used Statistical Analysis System (SAS)^®^ Studio, version 9.4, to compare absolute abundance of microbial species for time-of-wetness and original dust samples. Specifically, we used the PROC MULTTEST FDR Test Mean function in SAS. False Discovery Rate (FDR) was used instead of Positive False Discovery Rate (PFDR) due to the relatively small sample size ^49^. Species that did not occur in at least 10% of all samples were removed before analysis ^20,49,50^. All relative abundance data was transformed using the inverse hyperbolic sine function and combined with qPCR quantities to produce an absolute abundance value as previously described ^20^.

### Supplementary Information

Supplementary Information.

## Data Availability

The microbial sequencing data sets used in this study have been previously posted in publicly available databases. Earth-based sequences were previously reported ^20^ and are found in the European Nucleotide Archive, accession number PRJEB37053. All sequencing data from International Space Station samples were previously reported ^19^ in a recent study and can be found in the NASA GeneLab database, with identifiers 10.26030/87vb-2280, OSD-694, GLDS-623.
