# Supplementary material for: Fungal diversity differences in the indoor dust microbiome from built environments on earth and in space
Source: Sci Rep. 2024 May 24;14:11858. doi: 10.1038/s41598-024-62191-z (PMC11126634; doi:10.1038/s41598-024-62191-z)
Supplement: Supplementary file 1 — Supplementary Information. [file 41598_2024_62191_MOESM1_ESM.pdf]

Supplemental information for:

Fungal diversity differences in the indoor dust microbiome from built environments on Earth and in Space

Nicholas Nastasi[1,2,3], Sarah R. Haines[4], Ashleigh Bope[1,2,3], Marit E. Meyer[5], John M. Horack[6], and Karen C. Dannemiller[2,3,7*.]
[1]Environmental Science Graduate Program, Ohio State University, Columbus, OH 43210
[2]Department of Civil, Environmental, & Geodetic Engineering, College of Engineering, Ohio State University, Columbus, OH 43210
[3]Division of Environmental Health Sciences, College of Public Health, Ohio State University, Columbus, OH 43210
[4]Department of Civil and Mineral Engineering, University of Toronto, Toronto, Ontario, Canada, M5S 1A4
[5]NASA Glenn Research Center, Cleveland, OH 44135
[6]Department of Mechanical and Aerospace Engineering, College of Engineering and John Glenn College of Public Affairs, Ohio State University, Columbus, OH 43210
[7]Sustainability Institute, The Ohio State University, Columbus, OH 43210
*Corresponding Author Karen C. Dannemiller, Dannemiller.70@osu.edu, Civil, Environmental, and Geodetic Engineering; Environmental Health Sciences, The Ohio State University. 470 Hitchcock Hall, 2050 Neil Ave, Columbus, OH 43210. 614-292-4031.

Table S1: Adonis values for fungal beta diversity based on Bray-Curtis Dissimilarity between Earth-based residential house dust and dust collected from the International Space Station.

Variable	R^2	P-value
**Original Dust Samples only**		
Location (Earth vs. ISS)	0.232	0.001
**Fungal Time-of-Wetness Samples (All)**		
Location (Earth vs. ISS)	**0.347**	**0.001**
Time of Wetness	**0.015**	**0.003**
Day	**0.011**	**0.007**
**85% RH only**		
Location (Earth vs. ISS)	0.121	0.001
Time of Wetness	0.159	0.001
Day	0.007	0.793
**100% RH only**		
Location (Earth vs. ISS)	0.145	0.001
Time of Wetness	0.090	0.002
Day	0.025	0.086
**6-hour TOW only**		
Location (Earth vs. ISS)	0.398	0.001
Relative Humidity	0.013	0.438
Day	0.017	0.255
**12-hour TOW only**		
Location (Earth vs. ISS)	0.374	0.001
Relative Humidity	0.027	0.066
Day	0.008	0.816
**18-hour TOW only**		
Location (Earth vs. ISS)	0.175	0.001
Relative Humidity	0.185	0.001
Day	0.009	0.838
**24-hour TOW only**		
Location (Earth vs. ISS)	0.104	0.001
Relative Humidity	0.201	0.001
Day	0.008	0.786

Table S2: Fungal and bacterial alpha diversity comparisons between Earth-based and ISS dust for all conditions compared in this study.

Alpha Diversity Metrics			
**Fungi**			
**Condition**	**Location**	**Mean OTU**	**Mean Shannon Index**
Original Dust	Earth	465.2	5.11
	ISS	102.33	2.6
85% ERH 6-hours	Earth	516.91	5.59
	ISS	124.08	3.4
85% ERH 12-hours	Earth	416.2	5.13
	ISS	104.58	3.04
85% ERH 18-hours	Earth	517	5.28
	ISS	100.18	2.85
85% ERH 24-hours	Earth	397.6	4.97
	ISS	49	1.62
100% ERH 6-hours	Earth	374.11	5.25
	ISS	90.83	2.66
100% ERH 12-hours	Earth	403.27	5.04
	ISS	53.42	1.81
100% ERH 18-hours	Earth	405.58	4.61
	ISS	37.33	1.55
100% ERH 24-hours	Earth	240.5	3.67
	ISS	41.25	1.33
**Bacteria**			
Original Dust	Earth	236.5	6.72
	ISS	101.5	4.73
50, 85, 100% Day 14 24-hours	Earth	103.88	6.05
	ISS	52.56	5.04

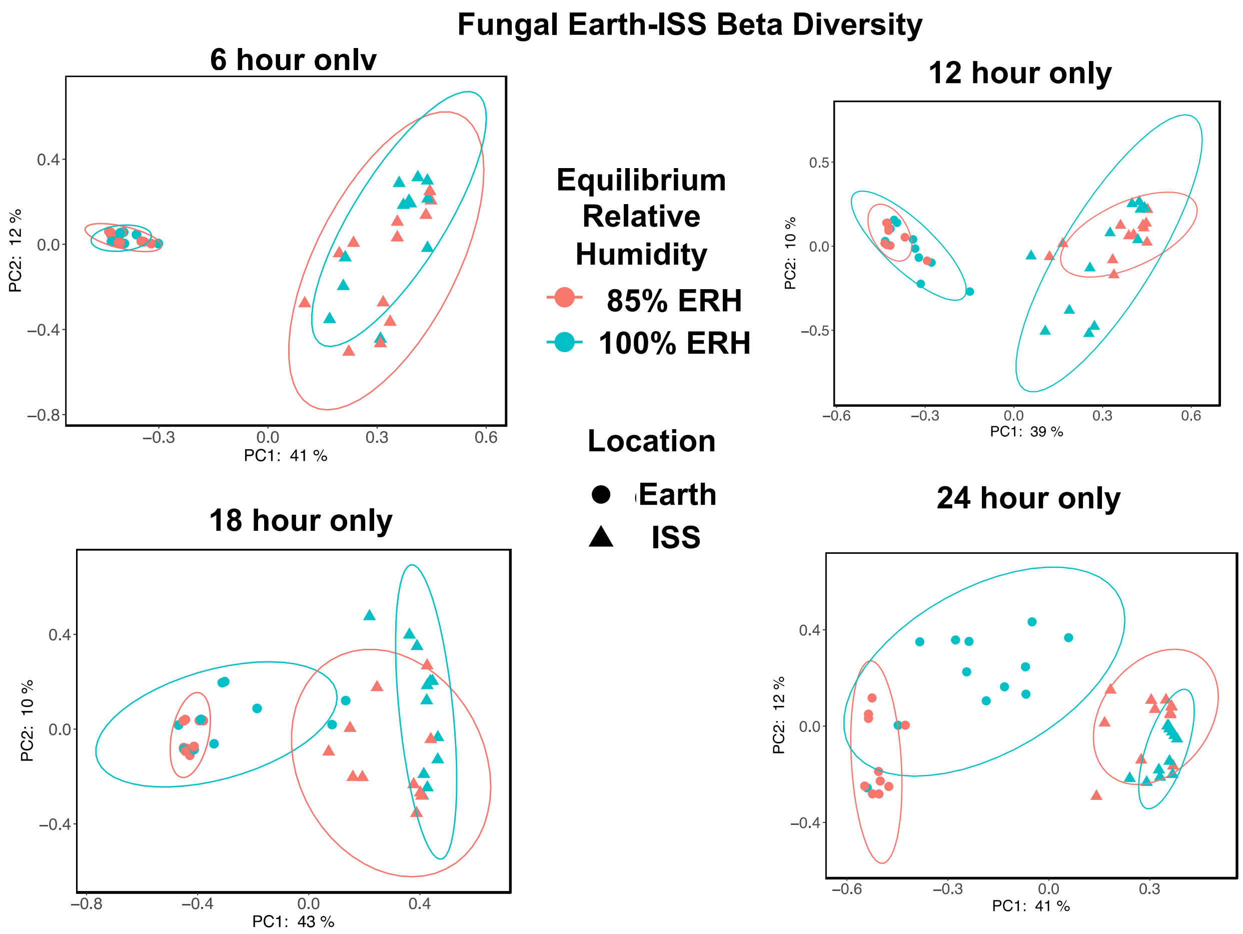

Figure S1: Fungal beta Bray Curtis dissimilarity principal coordinate analyses plots for time of wetness (TOW) samples separated out by each TOW value. For each plot, location of dust samples was indicated by shape (Earth – circle, ISS – triangle) while relative humidity conditions were indicated by color (85% - red, 100% blue). Ellipses represent 95% confidence interval for each set of data.

Table S3: Differential abundance comparison of fungal species between Earth-based house dust and ISS dust in original dust samples. A total of 791 fungal species were identified where 24 were significantly more abundant in ISS samples and 199 were more abundant in Earth-based house dust.

Differential Abundance Analysis Original Dust Samples (Earth-based vs ISS Dust)

Fungal Species	Unadjusted P-value	Adjusted FDR P-value
**More abundant on International Space Station**		
Aspergillus sydowii	<.0001	<.0001
Aspergillus unguis	<.0001	<.0001
Malassezia restricta	<.0001	<.0001

Malassezia globosa	<.0001	<.0001
Candida albicans	<.0001	<.0001
Rhodotorula mucilaginosa	<.0001	0.0011
Penicillium paczoskii	<.0001	0.0002
Cyberlindnera jadinii	<.0001	0.0006
Aspergillus ruber	<.0001	0.0011
Aspergillus flavus	<.0001	0.0023
Malassezia sympodialis	<.0001	0.0024
Aspergillus nidulans	<.0001	0.0027
Rhodosporidiobolus fluvialis	0.0002	0.0053
Aspergillus tamarii	0.0003	0.0061
Penicillium gladioli	0.0004	0.0076
Aspergillus falconensis	0.0008	0.0131
Penicillium concentricum	0.0019	0.0239
Fusarium acutatum	0.0027	0.026
Penicillium chrysogenum	0.0036	0.03
Candida tropicalis	0.0036	0.03
Malassezia arunalokei	0.0035	0.03
Malassezia dermatis	0.0041	0.0324
Papiliotrema laurentii	0.0054	0.0366
Aspergillus fischeri	0.0055	0.0366

More abundant in Earth-based House Dust *Only 24 out of 119 species shown

Neoascochyta desmazieri	<.0001	<.0001
Epicoccum brasiliense	<.0001	0.0001
Coniosporium apollinis	<.0001	0.0002
Rhodotorula babjevae	<.0001	0.0008
Pseudopithomyces chartarum	<.0001	0.0011
Pyrenochaetopsis pratorum	<.0001	0.0011
Vishniacozyma tephrensis	<.0001	0.0017
Xenodidymella humicola	<.0001	0.0019
Bipolaris sorokiniana	<.0001	0.0022
Pseudopithomyces rosae	<.0001	0.0025
Umbilicaria calvescens	<.0001	0.0027
Phaeosphaeria ampeli	<.0001	0.0031
Naganishia globosa	0.0001	0.0043
Curvibasidium pallidicoralli	0.0001	0.0043
Candida parapsilosis	0.0002	0.0053
Cystofilobasidium macerans	0.0002	0.0053
Cladosporium sphaerospermum	0.0002	0.0053
Articulospora proliferata	0.0002	0.0053
Preussia flanaganii	0.0002	0.0053
Monographella nivalis	0.0002	0.0054
Caloplaca ferrarii	0.0002	0.0057
Paracremonium inflatum	0.0003	0.007

Didymella nigricans	0.0004	0.0076
Knufia marmoricola	0.0004	0.008

Table S4: Kruskal-Wallis test statistics for fungal alpha diversity metrics comparing Earth-based residential house dust to dust collected from the ISS.

Fungal Alpha Diversity Kruskal-Wallis Statistics
Earth-based vs. ISS Dust

	Richness			Shannon Diversity		
**Condition**	**H**	**P-value**	**Q-value**	**H**	**P-value**	**Q-value**
**Original Dust Samples**						
Earth vs. ISS	18.44	< 0.0001	< 0.0001	17.20	< 0.0001	< 0.0001
**Fungal Time of Wetness (All Samples)**						
Earth vs. ISS	150.56	< 0.0001	< 0.0001	125.56	< 0.0001	< 0.0001
**Fungal Time-of-Wetness 85% ERH Only**						
Earth vs. ISS	65.27	< 0.0001	< 0.0001	58.04	< 0.0001	< 0.0001
**Fungal Time-of-Wetness 100% ERH Only**						
Earth vs. ISS	65.46	< 0.0001	< 0.0001	56.19	< 0.0001	< 0.0001
**Fungal Time-of-Wetness 6 hour Only**						
85% ERH, Earth vs. ISS	16.51	< 0.0001	< 0.0001	15.03	0.0001	0.0001
100% ERH, Earth vs. ISS	14.73	0.0001	0.0001	14.19	0.0002	0.0002
**Fungal Time-of-Wetness 12 hour Only**						
85% ERH, Earth vs. ISS	15.66	< 0.0001	< 0.0001	15.65	< 0.0001	< 0.0001
100% ERH, Earth vs. ISS	16.52	< 0.0001	< 0.0001	14.56	0.0001	0.0001
**Fungal Time-of-Wetness 18 hour Only**						
85% ERH, Earth vs. ISS	15.78	< 0.0001	< 0.0001	12.34	0.0004	0.0004
100% ERH, Earth vs. ISS	17.30	< 0.0001	< 0.0001	17.28	< 0.0001	< 0.0001
**Fungal Time-of-Wetness 24 hour Only**						
85% ERH, Earth vs. ISS	15.66	< 0.0001	< 0.0001	15.65	< 0.0001	< 0.0001
100% ERH, Earth vs. ISS	17.30	< 0.0001	< 0.0001	17.28	< 0.0001	< 0.0001

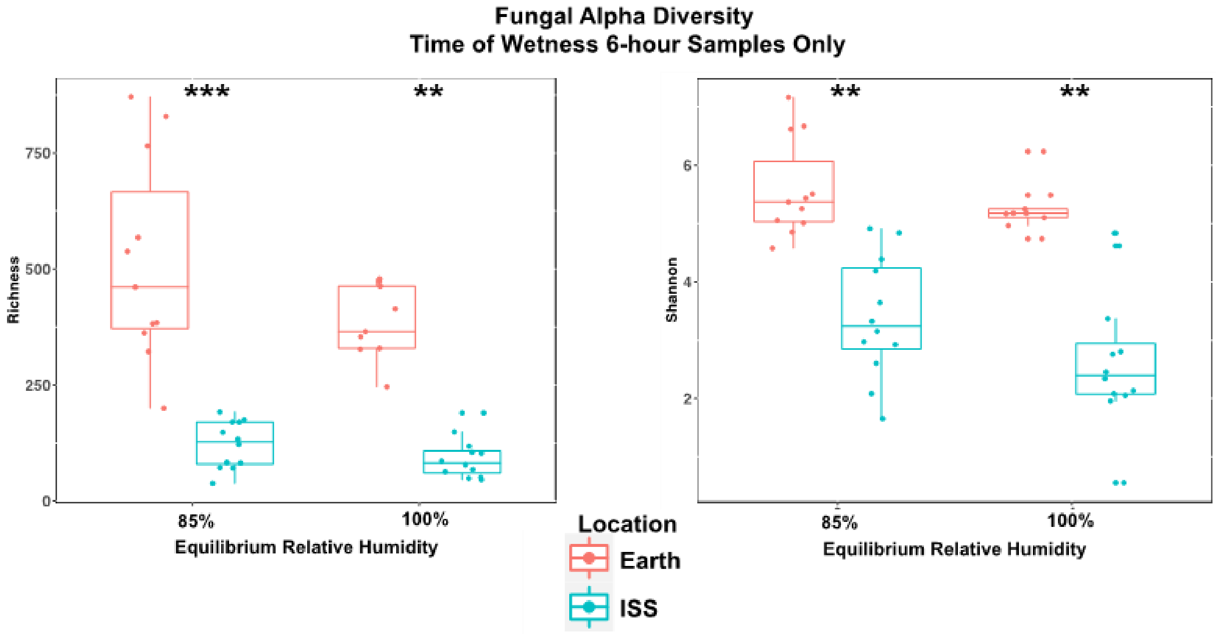

Figure S2: Fungal Richness and Shannon diversity plots for 6-hour time of wetness samples comparing relative humidity (RH) conditions (85% and 100% RH) for Earth-based house dust and dust collected from the ISS. Location is indicated by color (Earth – Red, ISS- Blue). ** Indicates Kruskal-Wallis t-test significance of P < 0.001, while *** indicates P < 0.0001.

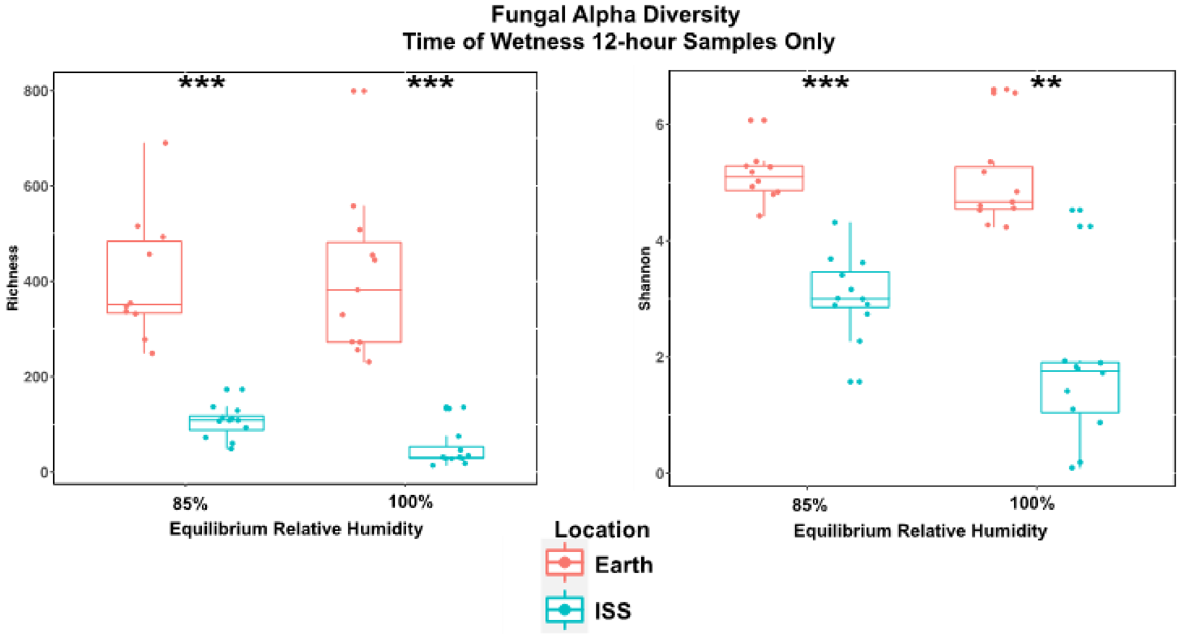

Figure S3: Fungal Richness and Shannon diversity plots for 12-hour time of wetness samples comparing relative humidity (RH) conditions (85% and 100% RH) for Earth-based house dust and dust collected from the ISS. Location is indicated by color (Earth – Red, ISS- Blue). ** Indicates Kruskal-Wallis t-test significance of P < 0.001, while *** indicates P < 0.0001.

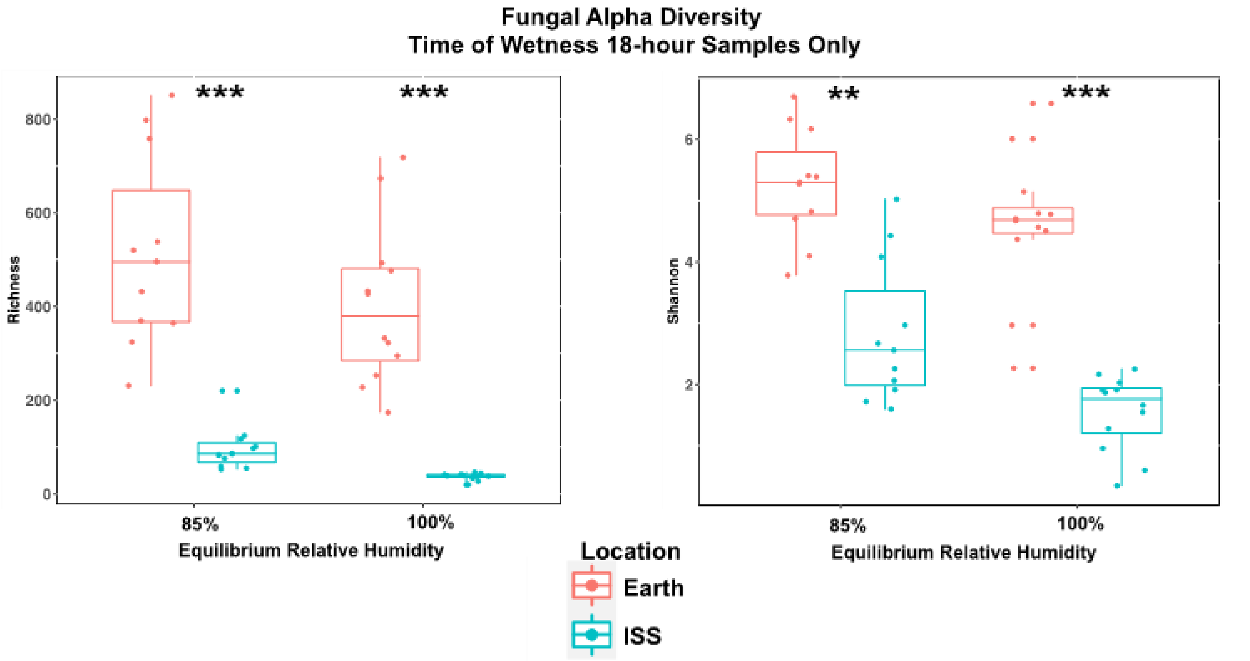

Figure S4: Fungal Richness and Shannon diversity plots for 18-hour time of wetness samples comparing relative humidity (RH) conditions (85% and 100% RH) for Earth-based house dust and dust collected from the ISS. Location is indicated by color (Earth – Red, ISS- Blue). ** Indicates Kruskal-Wallis t-test significance of P < 0.001, while *** indicates P < 0.0001.

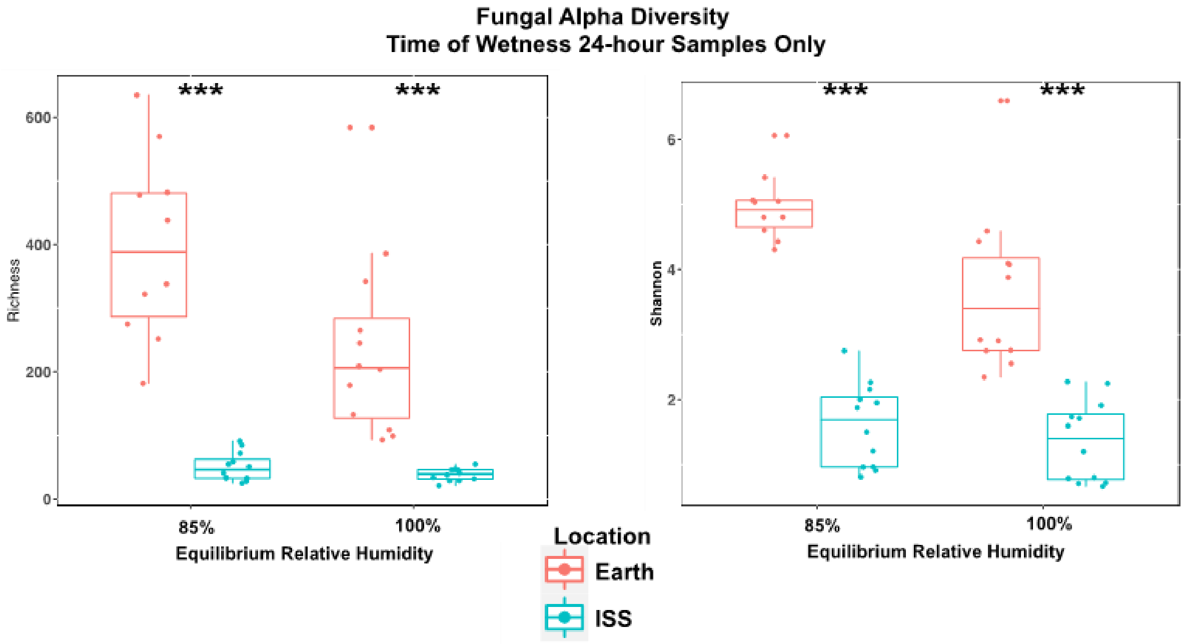

Figure S5: Fungal Richness and Shannon diversity plots for 24-hour time of wetness samples comparing relative humidity (RH) conditions (85% and 100% RH) for Earth-based house dust and dust collected from the ISS. Location is indicated by color (Earth – Red, ISS- Blue). *** Indicates Kruskal-Wallis t-test significance of P < 0.0001.

Table S5: Differential abundance comparison of fungal species between Earth-based house dust and ISS dust in all TOW conditions (6, 12, 18, and 24 hours) at 100% RH. A total of 521 fungal species were identified where 26 were significantly more abundant in ISS samples and 456 were more abundant in Earth-based house dust.

Differential Abundance Analysis TOW 100% RH Samples (Earth-based vs ISS Dust)

Fungal Species	Unadjusted P-value	Adjusted FDR P-value
**More abundant on International Space Station**		
Aspergillus sydowii	<.0001	<.0001
Aspergillus unguis	<.0001	<.0001
Aspergillus falconensis	<.0001	<.0001
Penicillium paczoskii	<.0001	<.0001
Candida albicans	<.0001	<.0001
Malassezia restricta	<.0001	<.0001
Malassezia globosa	<.0001	<.0001
Colletotrichum theobromicola	<.0001	<.0001
Aspergillus ruber	0.0001	0.0002
Aspergillus conicus	0.0001	0.0002
Malassezia sympodialis	0.0002	0.0002
Aspergillus tamarii	0.0001	0.0002
Lasiodiplodia margaritacea	0.0001	0.0002
Penicillium gladioli	0.0003	0.0005
Penicillium sclerotigenum	0.0003	0.0005
Rhodotorula mucilaginosa	0.0006	0.0008
Aspergillus nidulans	0.0005	0.0008
Malassezia dermatis	0.0008	0.0011

Penicillium atramentosum	0.0009	0.0012
Rhodosporidiobolus fluvialis	0.0011	0.0015
Saccharomyces cerevisiae	0.0029	0.0035
Aspergillus fischeri	0.0044	0.0051
Malassezia arunalokei	0.0046	0.0053
Aspergillus flavus	0.0051	0.0058
Trichothecium roseum	0.0071	0.008
Naganishia liquefaciens	0.0374	0.0406
**More abundant in Earth-based House Dust *Only 26 out of 456 species shown**		
Alternaria tenuissima	<.0001	<.0001
Fusarium culmorum	<.0001	<.0001
Cladosporium delicatulum	<.0001	<.0001
Mycosphaerella tassiana	<.0001	<.0001
Fusarium proliferatum	<.0001	<.0001
Nigrospora oryzae	<.0001	<.0001
Wallemia tropicalis	<.0001	<.0001
Echinoderma jacobi	<.0001	<.0001
Plectosphaerella oligotrophica	<.0001	<.0001
Aureobasidium namibiae	<.0001	<.0001
Cladosporium halotolerans	<.0001	<.0001
Vishniacozyma victoriae	<.0001	<.0001
Xenodidymella humicola	<.0001	<.0001

Cladosporium sphaerospermum	<.0001	<.0001
Pseudopithomyces chartarum	<.0001	<.0001
Neoascochyta desmazieri	<.0001	<.0001
Naganishia albida	<.0001	<.0001
Filobasidium magnum	<.0001	<.0001
Cystofilobasidium macerans	<.0001	<.0001
Coniosporium apollinis	<.0001	<.0001
Rhodotorula babjevae	<.0001	<.0001
Bipolaris sorokiniana	<.0001	<.0001
Phaeosphaeria ampeli	<.0001	<.0001
Epicoccum brasiliense	<.0001	<.0001
Trichosporon caseorum	<.0001	<.0001
Pseudopithomyces rosae	<.0001	<.0001

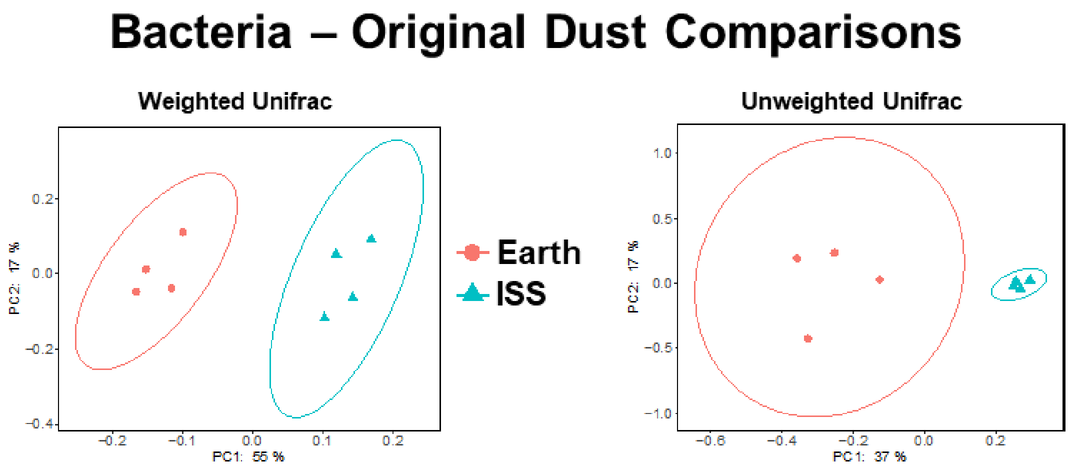

Figure S6: Bacterial beta diversity with Bray Curtis dissimilarity principal coordinate analyses plots for original dust samples. Location of dust samples are indicated by shape (Earth – circle, ISS – triangle). Ellipses represent 95% confidence interval for each set of data.

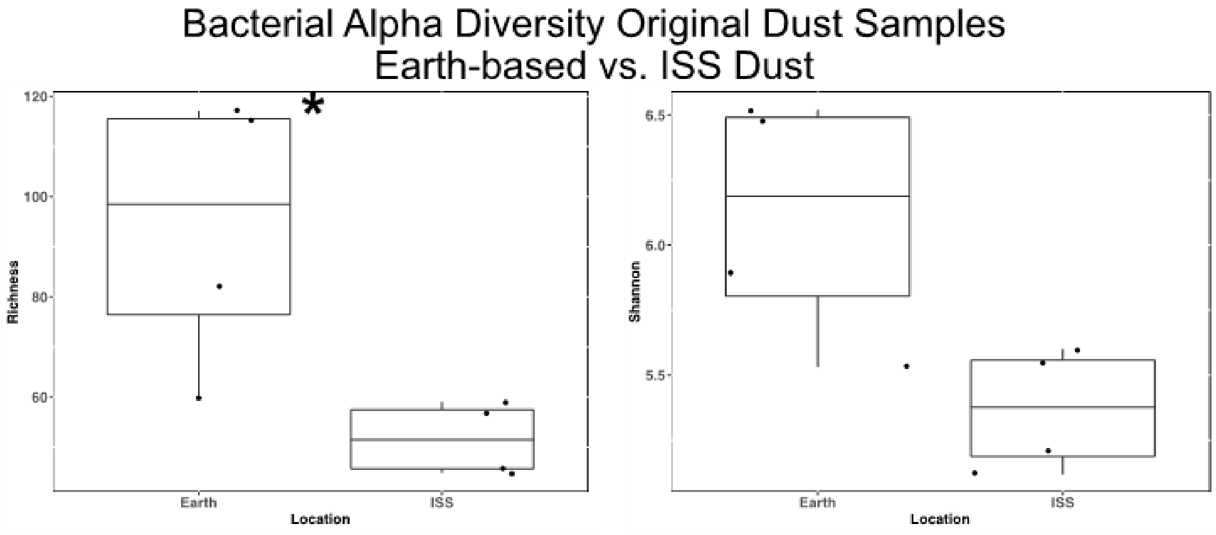

Figure S7: Bacterial Richness and Shannon diversity plots for original dust samples comparing Earth-based house dust to dust collected from the ISS. * Indicates Kruskal-Wallis t-test significance of P < 0.05.

Table S6: Kruskal-Wallis test statistics for bacterial alpha diversity metrics comparing Earth-based residential house dust to dust collected from the ISS.

	Richness			Shannon Diversity		
**Condition**	**H**	**P-value**	**Q-value**	**H**	**P-value**	**Q-value**
**Bacterial Original Dust Samples**						
**Earth vs. ISS**	**5.33**	**0.0209**	**0.0209**	**3.00**	**0.0832**	**0.0832**
**Bacterial Time-of-Wetness Samples**						
**Earth vs. ISS**	**11.38**	**0.0001**	**0.0001**	**9.79**	**0.0018**	**0.0018**

Table S7: Adonis values for bacterial beta diversity based on Bray-Curtis Dissimilarity between Earth-based residential house dust and dust collected from the International Space Station

	Unweighted Unifrac		Weighted Unifrac	
**Variable**	**R^2**	**P-value**	**R^2**	**P-value**
**Bacterial Time-of-Wetness (Original Dust, 50%, 85%, and 100% ERH 24-hours only)**				
**Location**	**0.221**	**0.001**	**0.159**	**0.004**
**ERH**	**0.079**	**0.002**	**0.343**	**0.001**
**Day**	**0.032**	**0.113**	**0.125**	**0.002**
**Original Dust Only**				
**Location**	**0.359**	**1**	**0.538**	**1**

Table S8: Differential abundance comparison of bacterial species between Earth-based house dust and ISS dust in original dust samples. A total of 81 bacterial species were identified where 3 were significantly more abundant in ISS samples and 11 were more abundant in Earth-based house dust.

Differential Abundance Analysis Original Dust Samples (Earth-based vs ISS Dust)

Bacterial Species	Unadjusted P-value	Adjusted FDR P-value
**More abundant on International Space Station**		
Acinetobacter rhizosphaerae	<.0001	0.0003
Prevotella melaninogenica	<.0001	0.0006
Rothia dentocariosa	0.0002	0.0021
**More abundant in Earth-based House Dust**		
Acinetobacter lwoffii	<.0001	0.0003
Flavobacterium succinicans	<.0001	0.0003
Methylobacterium adhaesivum	<.0001	0.0006
Paracoccus marcusii	<.0001	0.0006
Prevotella copri	<.0001	0.0006
Bacillus flexus	0.0318	0.1355
Eubacterium biforme	0.0305	0.1355
Pseudomonas viridiflava	0.0285	0.1355
Candidatus Nitrososphaera SCA1	0.0316	0.1355
Chloroidium saccharophilum	0.0316	0.1355
Candidatus Nitrososphaera	0.0336	0.1361
